# Clinical neutrophil-associated genes as reliable predictors of hepatocellular carcinoma

**DOI:** 10.3389/fgene.2022.989779

**Published:** 2022-10-06

**Authors:** Lishan Song, Chaojie Xu, Tong Zhang, Shengyang Chen, Shuiquan Hu, Bingbing Cheng, Hao Tong, Xiaoyong Li

**Affiliations:** The Fifth Affiliated Hospital of Zhengzhou University, Zhengzhou University, Zhengzhou, China

**Keywords:** hepatocellular carcinoma, WGCNA, neutrophils, risk score, tumor immune microenvironment

## Abstract

**Background:** Growing evidence suggests that infiltrating neutrophils are key players in hepatocellular carcinoma (HCC) tumor progression. However, a comprehensive analysis of the biological roles of neutrophil infiltration and related genes in clinical outcomes and immunotherapy is lacking.

**Methods:** HCC samples were obtained from the TCGA and GEO databases. The CIBERSORT algorithm was used to reveal the TIME landscape. Gene modules significantly associated with neutrophils were found using weighted gene co-expression network analysis (WGCNA), a “dynamic tree-cut” algorithm, and Pearson correlation analysis. Genes were screened using Cox regression analysis and LASSO and prognostic value validation was performed using Kaplan-Meier curves and receiver operating characteristic (ROC) curves. Risk scores (RS) were calculated and nomograms were constructed incorporating clinical variables. Gene set variation analysis (GSVA) was used to calculate signaling pathway activity. Immunophenoscore (IPS) was used to analyze differences in immunotherapy among samples with different risk scores. Finally, the relationship between RS and drug sensitivity was explored using the pRRophetic algorithm.

**Results:** 10530 genes in 424 samples (50 normal samples, 374 tumor samples) were obtained from the TCGA database. Using WGCNA, the “MEbrown” gene module was most associated with neutrophils. Nine genes with prognostic value in HCC (*PDLIM3*, *KLF2*, *ROR2*, *PGF*, *EFNB1*, *PDZD4*, *PLN*, *PCDH17*, *DOK5*) were finally screened. Prognostic nomograms based on RS, gender, tumor grade, clinical stage, T, N, and M stages were constructed. The nomogram performed well after calibration curve validation. There is an intrinsic link between risk score and TMB and TIME. Samples with different risk scores differed in different signaling pathway activity, immunopharmaceutical treatment and chemotherapy sensitivity.

**Conclusion:** In conclusion, a comprehensive analysis of neutrophil-related prognostic features will help in prognostic prediction and advance individualized treatment.

## 1 Introduction

Hepatocellular carcinoma (HCC) is the most common case type of liver cancer and the fifth leading cause of cancer-related death worldwide (Ioannou et al., 2007; Global Burden of Disease Study 2013 Collaborators, 2015; Lersritwimanmaen and Nimanong, 2018). Because symptoms are not obvious or asymptomatic in the early stages of cancer, most HCC cases are already in the incurable stage at the time of diagnosis. Even if detected early, with surgical resection, the best treatment, recurrence rates are high (Famularo et al., 2018; Fujiwara et al., 2018). This makes the prognosis of HCC poor (Jemal et al., 2017). Therefore, it is of great significance to study the influencing factors affecting HCC tumor progression and clinical prognosis, to develop novel and reliable indicators for treatment effect estimation, and to further advance individualized treatment.

In recent years, the progress of liver cancer treatment technology is encouraging, and many cutting-edge treatment methods have been discovered by researchers and applied in clinical practice. Among them, the development of anti-tumor immunotherapy is particularly rapid, and it has become a breakthrough in the treatment of liver cancer (Heinrich et al., 2018; Iñarrairaegui et al., 2018; Llovet et al., 2018). Immunotherapy is characterized by recognition by the immune system, and then by activating the immune system in the host to suppress or kill tumor cells, thereby reducing the rate of tumor recurrence and metastasis. International guidelines also clearly stated that immunotherapy is one of the effective methods for the treatment of advanced liver cancer (European Association for the Study of the Liver, 2018). Unfortunately, however, immunotherapy is only effective in a minority of HCC patients. The main reason for the limited nature of this treatment may be the suppression of the tumor immune microenvironment (TIME) (O'Reilly et al., 2019). TIME is a dynamic system composed of tumor cells and their surrounding immune cells, inflammatory cells, microvessels and various cytokines (Chew et al., 2017). TIME mainly affects the proliferation and metastasis of tumor cells by producing and activating cytokines, chemokines and growth factors, and recruiting immune cells (Yang et al., 2014). Among them, neutrophils play a particularly important role in this process.

As the most abundant leukocyte type in the human body, the anti-infection function of neutrophils has been generally recognized (Nathan, 2006; Nauseef and Borregaard, 2014). The important role of neutrophils in tumor cell progression and anti-tumor has long been confirmed by many studies (van Rees et al., 2016). There are many immune receptors on neutrophils that can bind to a variety of different extracellular ligands, thereby regulating activation and inhibition of signaling (van Rees et al., 2016). Among many cancer types, the role of neutrophils in HCC is particularly pronounced. Study finds that in HCC, circulating neutrophils may promote tumor development and can more accurately predict prognosis (Margetts et al., 2018; Quintela et al., 2019). In HCC tumor tissues, a high density of neutrophil infiltration is associated with shorter survival (Kuang et al., 2011). Recent studies have shown that neutrophils can be a potential therapeutic target for HCC (Geh et al., 2022). However, a comprehensive understanding of the role of neutrophils in the development of HCC is still lacking. The most reliable and efficient strategy for comprehensively assessing tumor susceptibility to clinical therapy is likely to be derived from the immune profile. Therefore, it is of great interest to identify HCC cases based on neutrophil-related risk profiles, thereby facilitating individualized treatment.

In this paper, we explore the potential role of neutrophils using the TCGA dataset, with external validation using the GEO (Gene Expression Omnibus) dataset. The abundance of 22 tumor-infiltrating immune cells (TIC) subtypes was obtained using the CIBERSORT algorithm. Gene modules significantly associated with neutrophils were identified by weighted gene coexpression network analysis (WGCNA). From these 590 genes, we finally got 9 genes significantly associated with HCC. The risk score (RS) was calculated according to the contribution of each gene to prognosis, and all samples were grouped according to the median RS. Subsequently, based on risk characteristics and other clinical variables, we developed and validated a HCC prognostic nomogram. We explored the synergistic effects of RS and tumor mutational burden (TMB) and potential relationships with TIME and cell signaling pathways. Finally, the effect of risk characteristics on the efficacy of immunotherapy and chemotherapy was investigated. In conclusion, based on neutrophil-related genes, reliable biological indicators and prognostic indicators for predicting the clinical prognosis of HCC have been established, which can guide for the precise treatment of HCC.

## 2 Materials and methods

### 2.1 Data download and preprocessing

The TCGA-LIHC dataset was downloaded from the TCGA portal and 424 HCC samples were obtained. There are 374 tumor samples and 50 normal tissue sequencing profiles. Among the 374 tumor samples, four samples had missing clinical data, and the remaining 370 tumor samples were used for follow-up studies. We obtained somatic mutation data from the TCGA database and analyzed copy number variation (CNV) to further analyze potential relationships between neutrophil-related risk signatures and TMB. The GSE76427 cohort was obtained from the GEO database for external validation.

### 2.2 Landscape of immune cell infiltration

The sequencing data of the samples were analyzed using the CIBERSORT algorithm (http://cibersort.stanford.edu/) to obtain the relative abundance of 22 TICs subtypes. The relative abundance of these TICs can be used to represent the structural composition of TIME cells.

### 2.3 Neutrophil-associated gene module

We selected 10,530 genes from TCGA-LIHC as data and the relative abundance of 22 TICs subtypes as the phenotype of interest. We used WGCNA to study the association between gene co-expression networks and phenotypes (Langfelder and Horvath, 2007; Langfelder and Horvath, 2008; Gysi et al., 2018). A “dynamic tree-cut” algorithm was used to introduce similar genes into the same candidate module. Pearson correlation test (*p* < 0.05) was used to analyze the correlation between the module eigengenes and the phenotype of interest. Finally, we focused on the “neutrophil” population and extracted the gene modules most significantly associated with neutrophils for subsequent analysis.

### 2.4 Construction of neutrophil-related prognostic features

To investigate the impact of neutrophil-related genes on the prognosis of HCC cases, we extracted 590 genes in the “MEbrown” gene module. Through univariate Cox regression analysis, Lasso regression and multivariable Cox regression analysis, 9 neutrophil-related genes (*PDLIM3*, *KLF2*, *ROR2*, *PGF*, *EFNB1*, *PDZD4*, *PLN*, *PCDH17*, *DOK5*) related to the prognosis of HCC were finally screened. The TCGA cohort was used as our training set, and the risk score (RS) was calculated as follows: ↓

$$riskscore=\overset{n}{\underset{i=1}{\sum }}\left(coefi*Xi\right)$$

### 2.5 Validation of prognostic neutrophil-related features

RS was calculated for each HCC sample. Taking the median of RS as the dividing line, all samples were divided into two parts: high-risk group (HRG) and low-risk group (LRG). Kaplan-Meier (KM) survival analysis was used and the KM survival curve was drawn to compare the survival difference between the HRG and LRG. To verify its prognostic value, we mapped the transient receiver operating characteristic (ROC). Univariate Cox regression and multivariate Cox regression were used to validate RS as an independent prognostic factor for HCC patients.

### 2.6 Establishment and verification of nomogram

To intuitively and accurately predict the 1-year, 3-year, and 5-year survival probability of HCC patients, we combined RS with clinical variables to draw a prognostic nomogram. Calibration curves were used to verify the performance of the model.

### 2.7 Gene set enrichment analysis

The c2.cp.kegg.v7.4.symbols collection were used to explore the function annotation by GSEA software.

### 2.8 Relationship between tumor mutational burden and risk score

Somatic mutation data were obtained from the TCGA database. Waterfall plots of HRG and LRG were drawn using the “maftools” R package (Yoshihara et al., 2013). Differences in the survival of HCC patients were analyzed according to median TMB and RS.

### 2.9 Association of tumor immune microenvironment and risk score

To investigate the potential association between RS and TIME, we assessed immune infiltration using seven methods. Immune cells and stromal cells in HCC malignancies were estimated using the ESTIMATE algorithm (Yoshihara et al., 2013). We calculated immune and stromal scores and explored the relationship between RS and immune infiltration signatures by the Spearman correlation.

### 2.10 Genome variation analysis

To assess the activation of hallmark and metabolic pathways described in the MSigDB database (Chan et al., 2019), we used the GSVA package (version 1.36.3) to pass the Gene Set Variation Analysis (GSVA) (Rizvi et al., 2015) predicts pathway activity and ultimately assesses relative pathway activity in a single sample.

### 2.11 Predicting the effect of immunotherapy

To explore the potential association between immunotherapy and RS, we analyzed the association between the expression levels of immune checkpoint blockade-related genes (PDCD1, etc.) and HRG/LRG. In this process, Immunophenoscore (IPS) (Charoentong et al., 2017) was used as a novel robust predictor of response to immunotherapy regimens.

### 2.12 Prediction of chemotherapy effect

To explore the relationship between RS and drug sensitivity, we constructed a ridge regression model based on the Genomics of Drug Sensitivity in Cancer cell lines and TCGA gene expression profiles. Using the pRRophetic algorithm, half-maximal inhibitory concentrations (IC50) were estimated for four chemotherapeutic agents (sorafenib, gemcitabine, cisplatin, and doxorubicin) in HCC patients.

### 2.13 Statistical analysis

The Wilcoxon test and the Kruskal–Wallis test were used to compare two groups and more than two groups of variables, respectively. The analysis of RS and TMB was performed by the chi-square test, and the correlation between coefficients was analyzed by Spearman. Two-sided *p* < 0.05 was considered statistically significant. All statistical calculations were done in R software (version 4.1.1).

## 3 Results

### 3.1 Tumor immune microenvironment landscape in hepatocellular carcinoma

Table 1 lists the data characteristics of the samples in this study after preprocessing. The number of complete follow-up data samples available for the TCGA-LIHC and GSE76427 datasets is 370 and 115, respectively. The median follow-up time for the two cohorts was 1.66 and 1.16 years, respectively. The probabilities of end-point events in the two cohorts were 35.68% and 20.00%, respectively.

**TABLE 1:** Clinicopathological characteristics of HCC patients from the TCGA and GSE76427 databases.

Characteristics	TCGA-LIHC cohort	GSE76427
	N = 370	N = 115
**Age**		
<=65	229 (61.89%)	65 (56.52%)
>65	141 (38.11%)	50 (43.48%)
Gender		
Female	119 (32.16%)	22 (19.13%)
Male	251 (67.84%)	93 (80.87%)
**Grade**		
1–2	232 (62.70%)	NA
3–4	133 (35.95%)	NA
Unknow	5 (1.35%)	NA
**Stage**		
I–II	258 (69.73%)	NA
III–IV	88 (23.78%)	NA
Unknow	24 (6.49%)	NA
**T**		
T0–T2	276 (74.59%)	NA
T3–T4	92 (24.86%)	NA
Unknow	2 (0.54%)	NA
**M**		
M0	367 (99.19%)	NA
M1	3 (0.81%)	NA
**N**		
N0–N1	365 (98.65%)	NA
N2–N3	4 (1.08%)	NA
Unknow	1 (0.27%)	NA
**Survival status**		
Alive	238 (64.32%)	92 (80.00%)
Dead	132 (35.68%)	23 (20.00%)
**The median follow-up time (year)**	1.66	1.16

**FIGURE 1:**
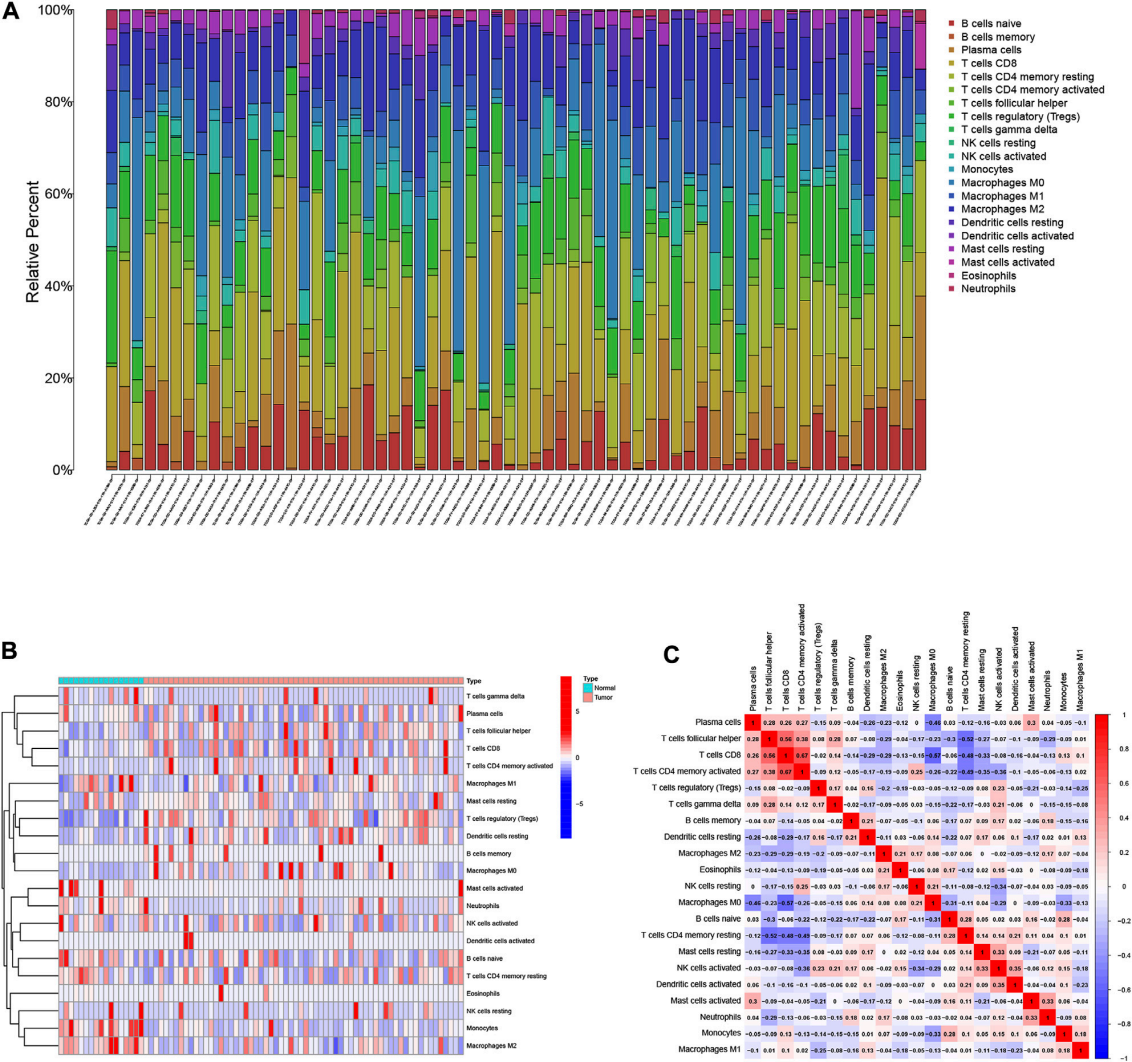
Landscape of immune cell infiltration in the tumor immune environment of HCC. Subpopulation of 22 immune cell subtypes **(A)** and proportional heatmap of 22 TICs in each HCC samples **(B)**. **(C)** Intrinsic correlation of 22 infiltrating immune cells in HCC.

The relative abundances of the 22 TICs isoforms (Figure 1A) were obtained using the CIBERSORT algorithm as shown in Supplementary Table S1. The comprehensive heatmap we created (Figure 1B) visualized the differences in the TIME landscape between tumor and normal tissues. Potential connections between various TIME immune cells in HCC tissues are shown in Figure 1C. Neutrophils were positively correlated with B cells memory (r = 0.18, *p* < 0.05) and significantly negatively correlated with T cells follicular helper (r = -0.29, *p* < 0.05).

### 3.2 Establish the weighted gene co-expression network analysis network

**FIGURE 2:**
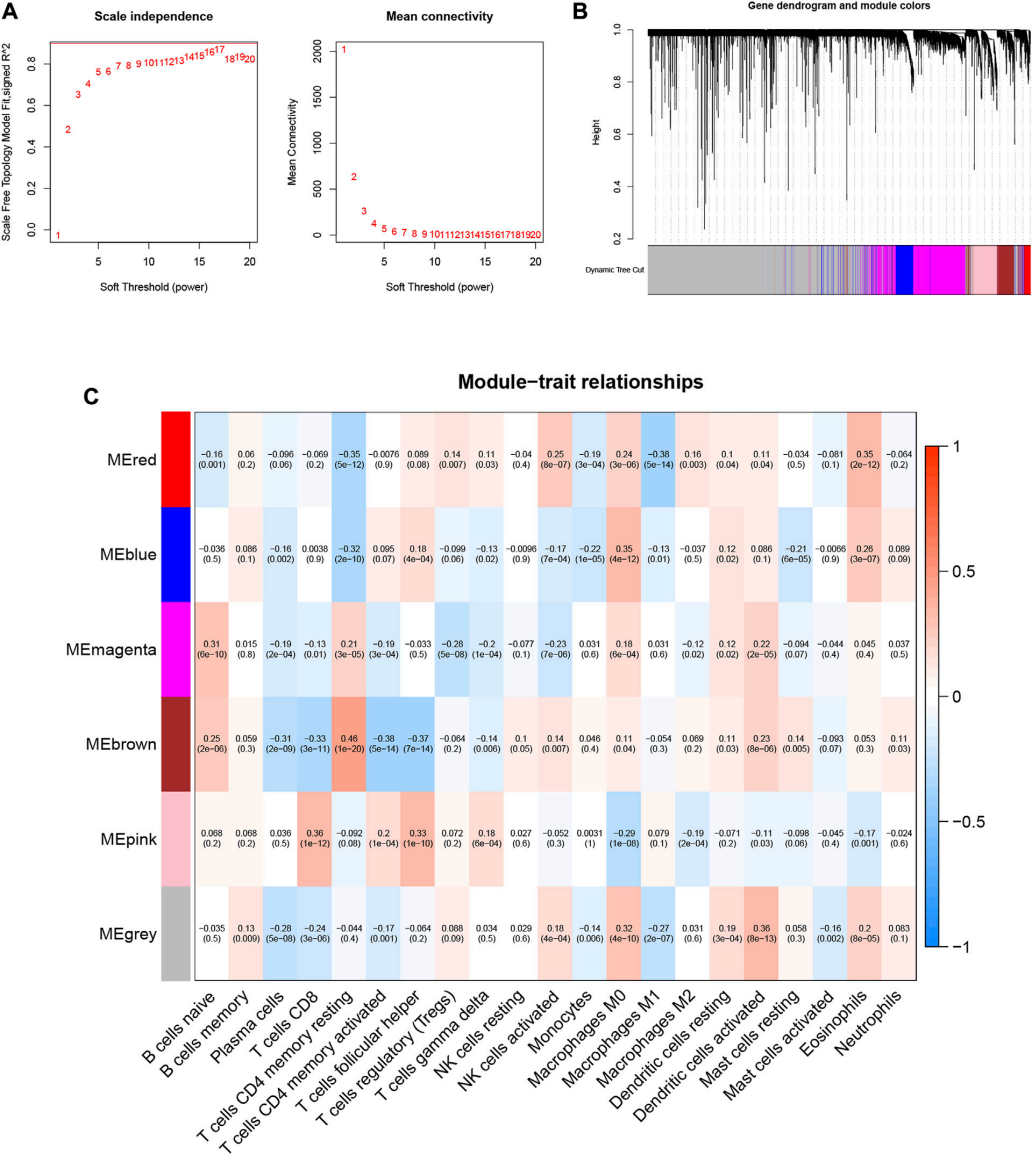
Choosing an appropriate soft threshold (power) and building a hierarchical clustering tree. **(A)** The choice of the soft threshold enables the scale-free topology to achieve an exponent of 0.90, and the average connectivity for 1–20 soft threshold powers is analyzed. **(B)** Neutrophil-related genes with similar expression patterns were merged into the same module using a dynamic tree-cutting algorithm, creating a hierarchical clustering tree. Heatmap of correlations between **(C)** modules and immune-infiltrating cells (traits).

10,530 gene data and immune-infiltrating subsets were extracted from the TCGA-LIHC dataset to develop the WGCNA network. The scale-free network was constructed by setting the optimal soft-threshold power (β) to the first power value of 17 when the scale-free topology index reached 0.90 (Figure 2A). Weighted hierarchical clustering analysis was then performed and the results were segmented, resulting in 6 gene modules (Figure 2B). The relationship between each immune cell and candidate gene modules in HCC tumor tissues was analyzed using Pearson correlation, and the results are shown in Figure 2C. By observation, we can easily find that the module with the strongest correlation with neutrophils is “MEbrown” (r = 0.11, *p* = 0.03). We used the 590 genes in the “MEbrown” module (Supplementary Table S2) for further analysis.

### 3.3 Development risk signature

**FIGURE 3:**
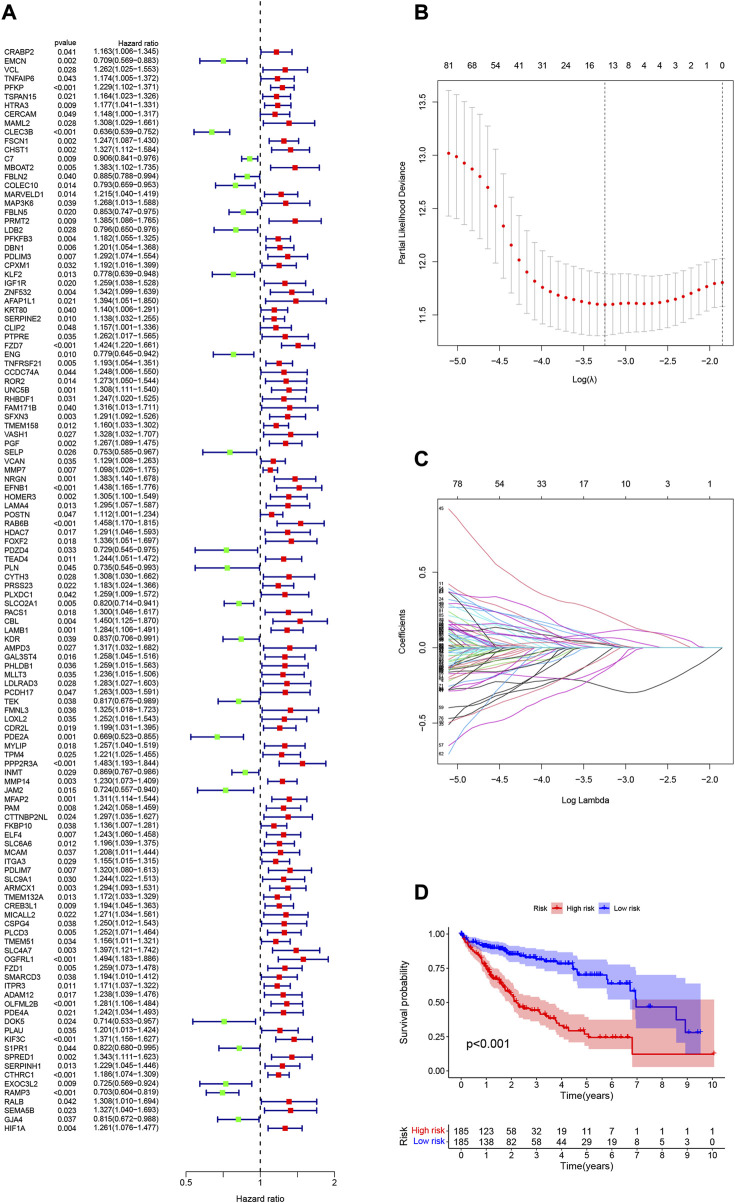
**(A)** 122 genes associated with HCC prognosis screened using univariate Cox regression. **(B)** Variation curve of regression coefficient with Log (λ) in Lasso regression. **(C)** Ten-fold cross-validation for tuning parameter selection in lasso regression.Vertical lines are drawn from the best data according to the minimum criterion and 1 standard error criterion. **(D)** Kaplan-Meier curve analysis showed differences in overall survival between high- and low-risk groups in the TCGA-LIHC cohort.

Neutrophil-related gene expression data and prognostic information of HCC patients were extracted from the TCGA-LIHC dataset. Using univariate Cox regression analysis, 122 genes were initially screened from 590 genes (*p* < 0.05, Supplementary Table S3). To more intuitively show the results of univariate Cox regression, we draw Figure 3A. To prevent overfitting, we performed a lasso regression analysis on the 122 genes obtained above (Figures 3B,C). Finally, through multivariate Cox regression analysis, we screened out 9 neutrophil-related genes (*PDLIM3*, *KLF2*, *ROR2*, *PGF*, *EFNB1*, *PDZD4*, *PLN*, *PCDH17*, *DOK5*, Supplementary Table S4) that are beneficial for predicting the prognosis of HCC patients. The RS was computed: risk score(RS)=(0.2945×PDLIM3)−(0.4333×KLF2)+(0.2322×ROR2)+(0.5494×PGF)+(0.1935×EFNB1)−(0.4964×PDZD4)−(0.4623×PLN)+(0.3700×PCDH17)−(0.4628×DOK5)

The 370 HCC samples were divided into HRG and LRG according to median RS to reveal potential optimal values for neutrophil-related genes.

### 3.4 Validation of prognostic risk characteristics

**FIGURE 4:**
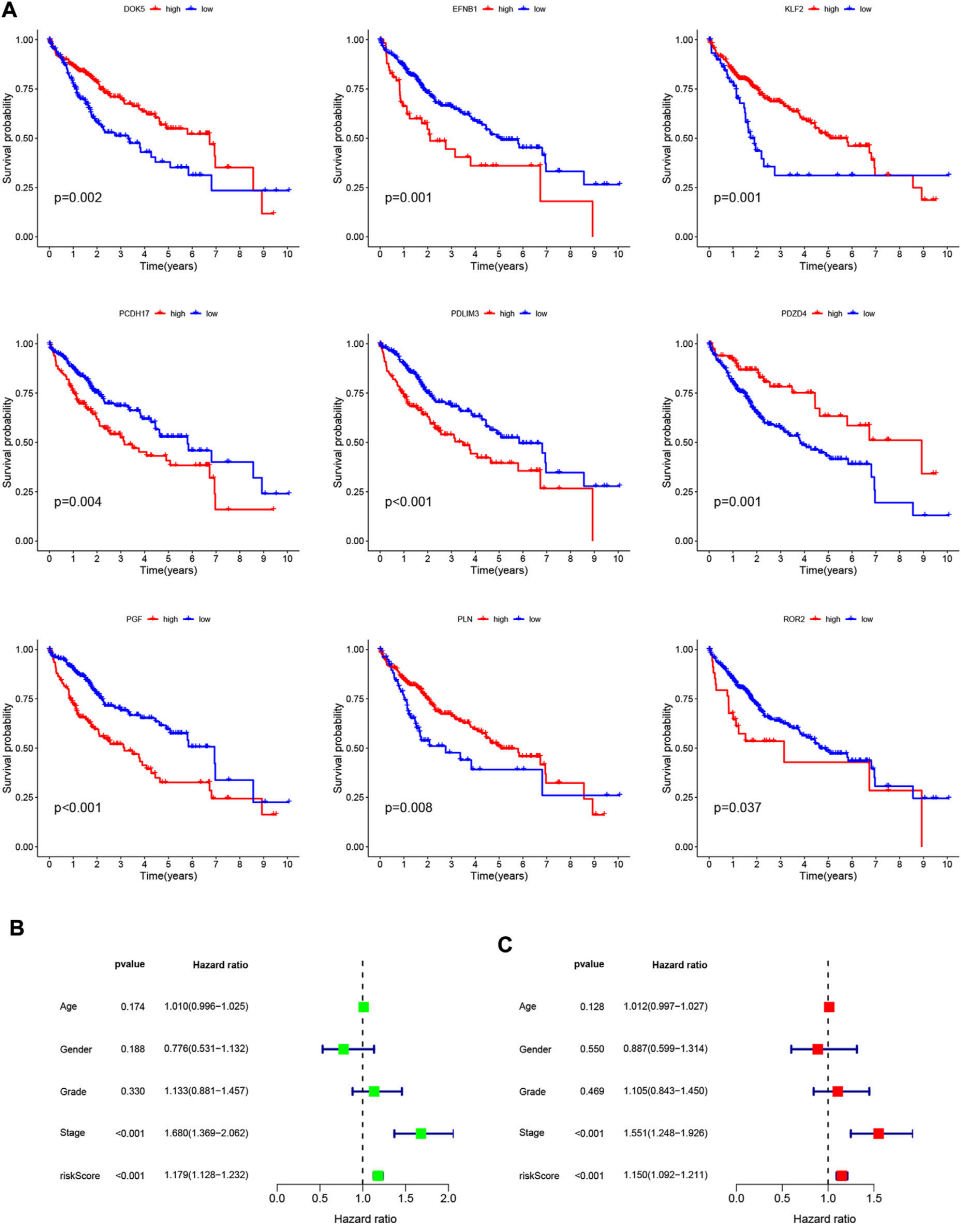
**(A)** Kaplan-Meier curve analysis showed the difference in overall survival between the high and low expression groups for 9 neutrophil-related genes (*PDLIM3*, *KLF2*, *ROR2*, *PGF*, *EFNB1*, *PDZD4*, *PLN*, *PCDH17*, *DOK5*). **(B)** Univariate Cox regression results for overall survival. **(C)** Multivariate Cox regression results for overall survival.

**FIGURE 5:**
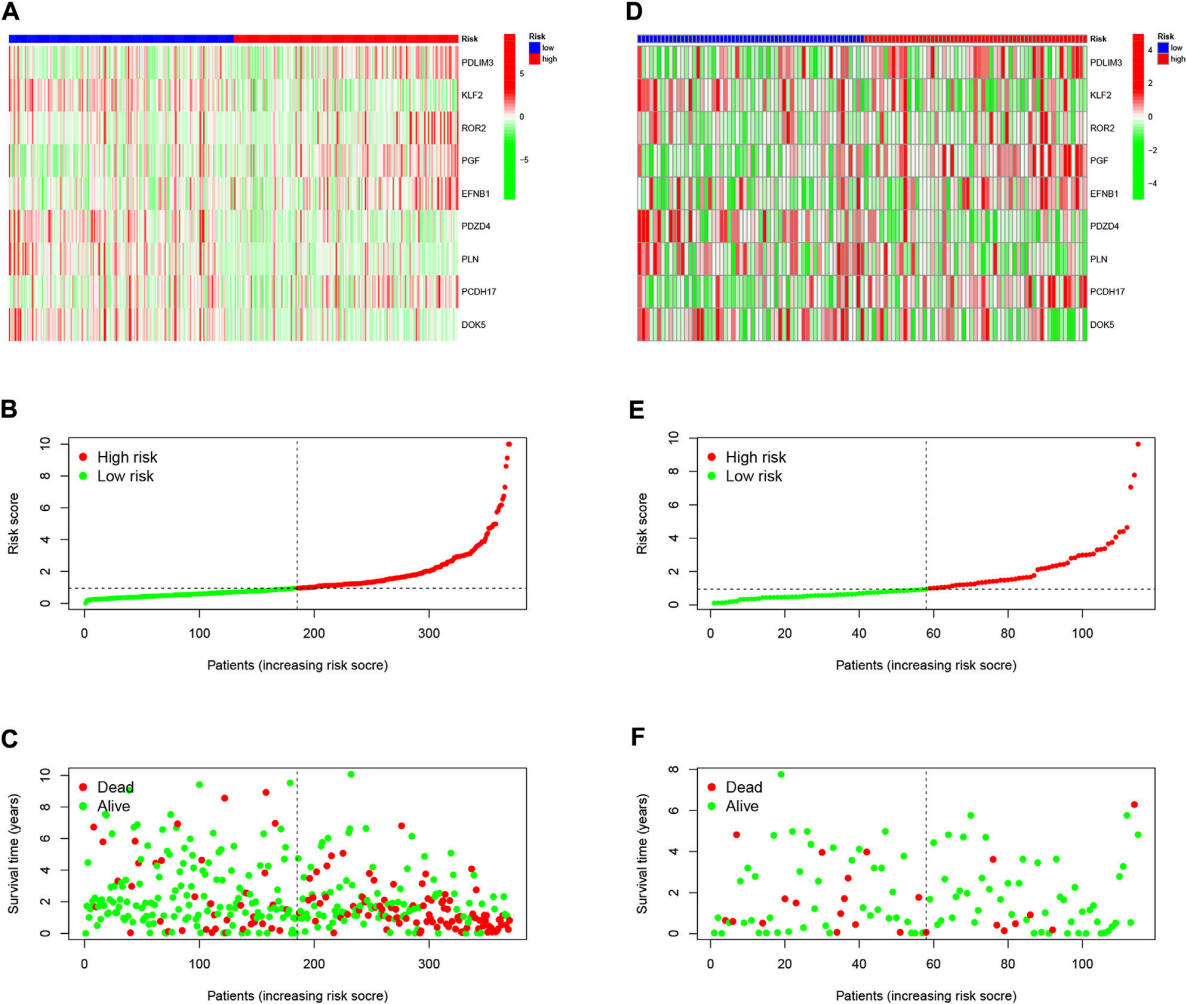
**(A)** Confirmation of prognostic risk scores in the TCGA cohort. **(B)** Polygenic model risk score distribution in the TCGA cohort. **(C)** Survival status and duration of HCC patients in the TCGA cohort. **(D)** Confirmation of prognostic risk scores in the GSE76427 cohort. **(E)** Polygenic model risk score distribution in the GSE76427 cohort. **(F)** Survival status and duration of LUAD patients in the GSE76427 cohort.

To validate the scientific validity of the risk signature, we used 370 HCC samples in the TCGA-LIHC dataset for internal validation. The KM survival curve we plotted indicated that HRG had a poor prognosis (*p* < 0.001, Figure 3D). In addition, we performed an external validation of the survival results using the GSE76427 dataset, the results of which are shown in Supplementary Figure S1. All samples were regrouped according to the median expression of each gene. The KM survival curve was drawn with the expression of a single gene as the only variable. The results showed that each neutrophil-related gene had a significant impact on the clinical prognosis of HCC (*p* < 0.05, Figure 4A). We combined RS with clinical variables such as gender and age, and explored the potential role of RS in predicting the prognosis of HCC. The hazard ratios (HR) for RS in univariate Cox regression and multivariate Cox regression were 1.179 (95% CI 1.128–1.232; Figure 4B) and 1.150 (95% CI 1.092–1.211; Figure 4C), respectively. These results suggest that the risk signature developed based on neutrophil-related genes has good prognostic predictive power and can serve as an independent risk factor for the prognosis of HCC patients. The expression patterns of 9 genes, the distribution of sample survival status, and the corresponding risk scores among the 370 samples in the TCGA-LIHC cohort are shown in Figures 5A–C. In addition to this, we also performed external validation using 115 independent samples from the GSE76427 cohort (Figures 5D–F). These results all clearly demonstrate that neutrophil-related risk-prognostic features have stable and robust prognostic value.

### 3.5 Functional analysis of neutrophil-related genes

**FIGURE 6:**
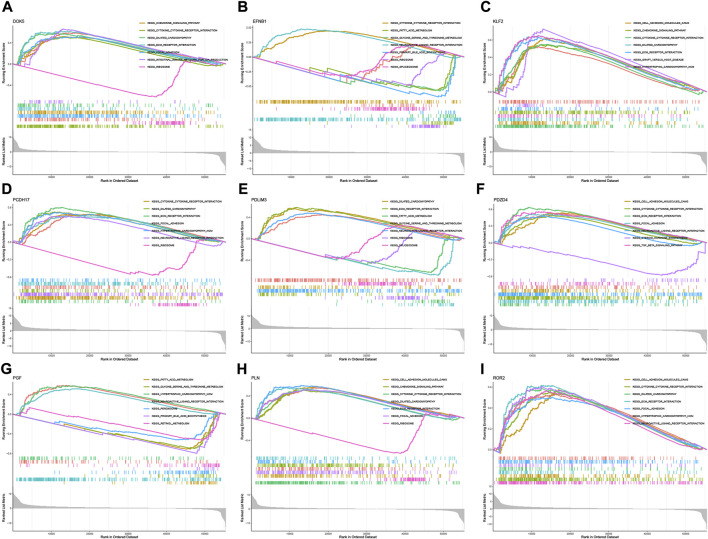
GSEA for samples with high and low expression of 9 central genes. **(A)** Enriched gene set collected in KEGG for samples with high *DOK5* expression. **(B)** Enriched gene set collected in KEGG for samples with high *EFNB1* expression. **(C)** Enriched gene set collected in KEGG for samples with high *KLF2* expression. **(D)** Enriched gene set collected in KEGG for samples with high *PCDH17* expression. **(E)** Enriched gene set collected in KEGG for samples with high *PDLIM3* expression. **(F)** Enriched gene set collected in KEGG for samples with high *PDZD4* expression. **(G)** Enriched gene set collected in KEGG for samples with high *PGF* expression. **(H)** Enriched gene set in the KEGG collection for samples with high *PLN* expression. **(I)** Enriched gene set in the KEGG collection for samples with high *ROR2* expression.

According to the median expression level of each neutrophil-related gene, we divided all samples into high and low expression groups. Then, high and low gene expression groups were functionally enriched using GSEA (Figures 6A–I). KEGG enrichment analysis showed that the high expression of the *EFNB1* gene was related to cytokine cytokine receptor interaction. The high expression of the *PGF* gene was significantly enriched in signaling pathways such as hypertrophic cardiomyopathy, neuroactive ligand-receptor interaction and fatty acid metabolism.

### 3.6 Distribution of clinical variables and risk scores in samples

**FIGURE 7:**
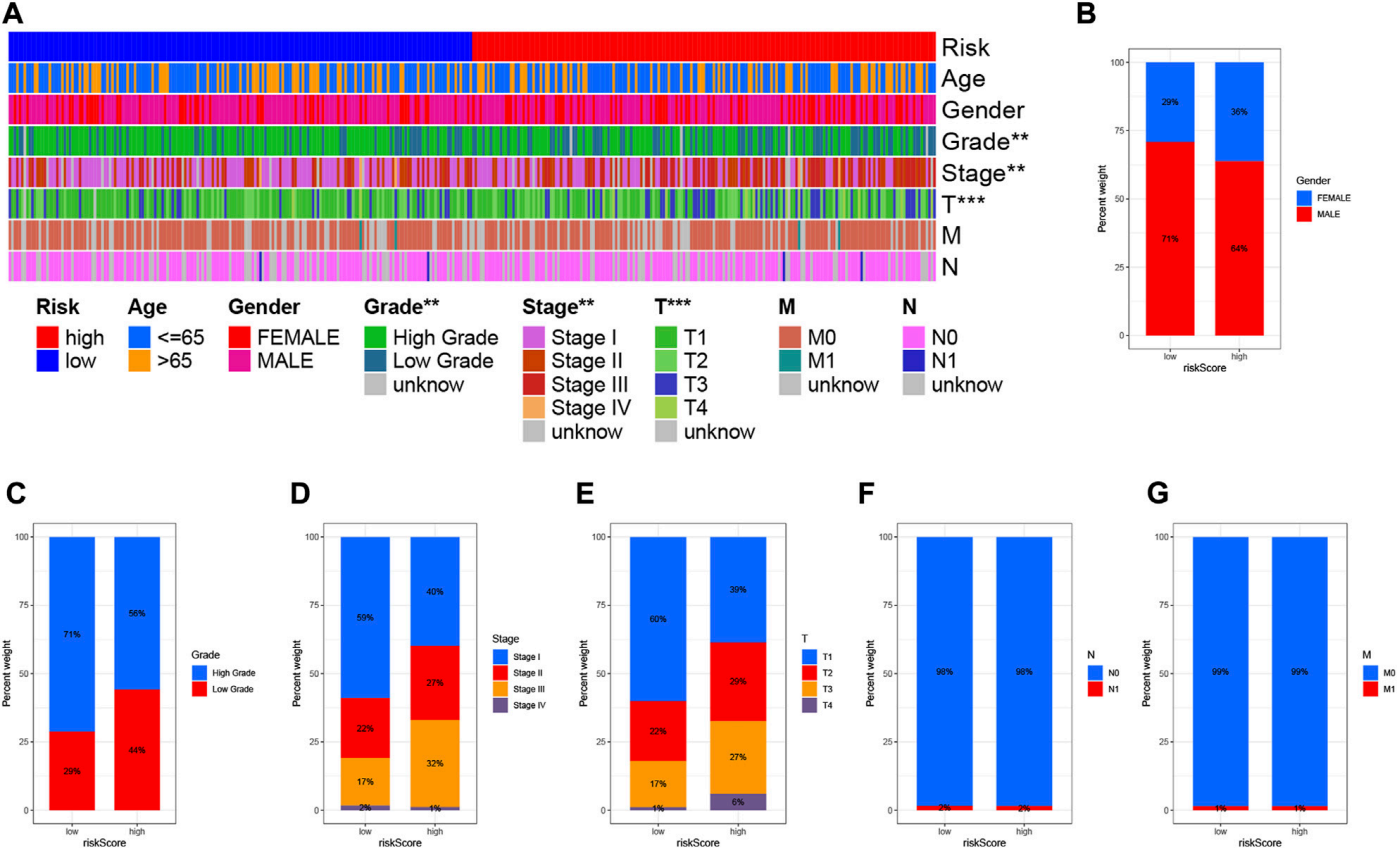
Clinical significance of prognostic risk characteristics. **(A)** Heatmap showing the distribution of clinical characteristics and corresponding risk scores in each sample. Incidence of clinical variable subtypes of LRG/HRG. **(B)** Gender, **(C)** Grade, **(D)** Clinical stage, **(E)** Stage T, **(F)** Stage N, and **(G)** Stage M.

To visualize the distribution of clinical variables in low/high risk subgroups, we plotted Figure 7A. The proportions of gender, WHO grade, clinical stage, T, N, and M stage clinical subtypes in the low/high risk subgroup are shown in Figures 7B–G.

### 3.7 Construction of prognostic nomogram

**FIGURE 8:**
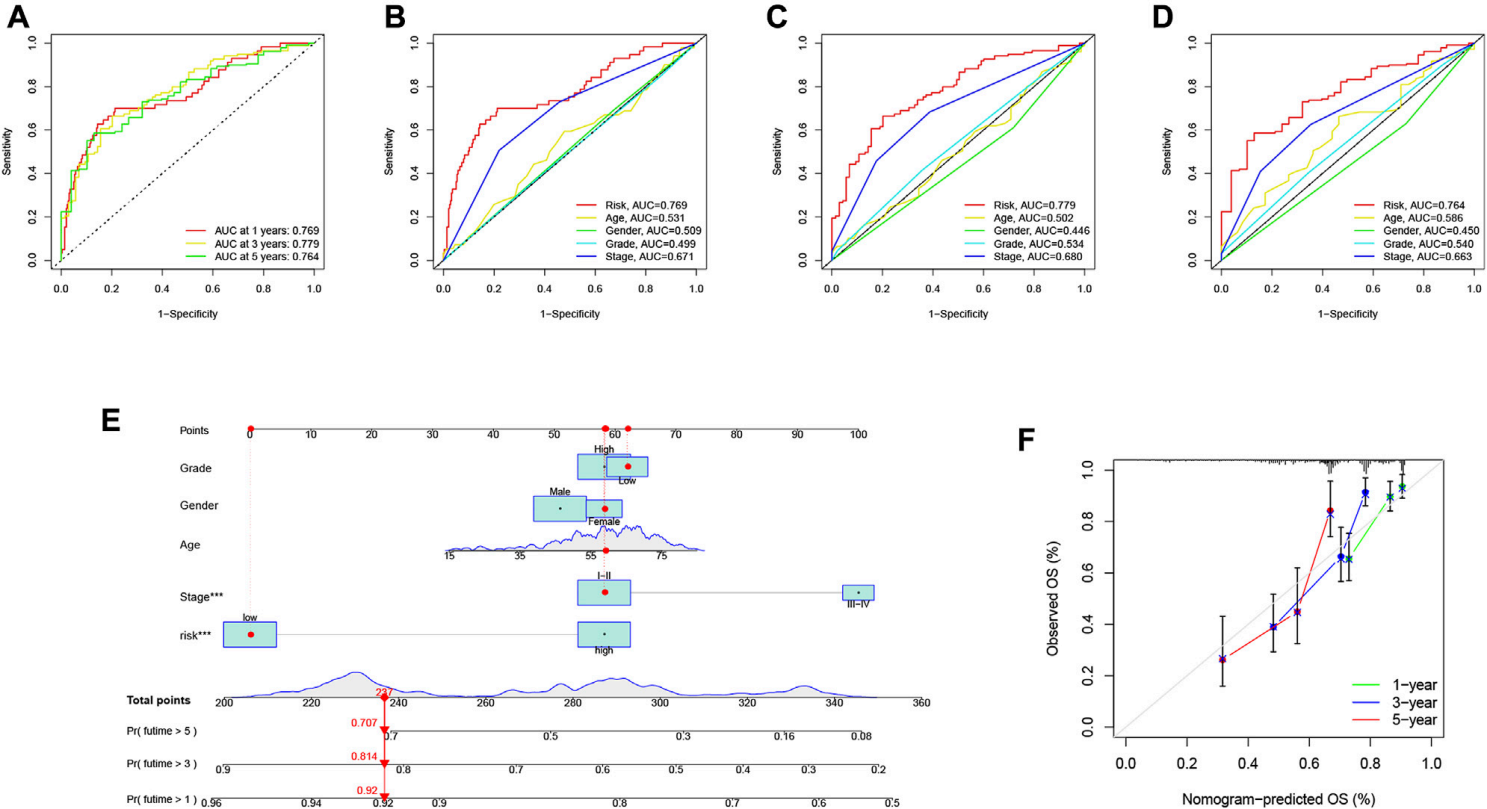
Validation of prognostic efficiency of risk signatures. **(A)** ROC analysis was used to estimate the predictive value of prognostic features. **(B–D)** The area under the curve (AUC) of the risk score for predicting overall survival at 1, 3, and 5 years and other clinical characteristics. **(E)** Nomogram was used to predict survival in HCC patients. **(F)** 1-, 3-, and 5-year nomogram calibration curves.

The area under the curve (AUC) of the ROC curve we drew was 0.769, 0.779 and 0.764 for 1-year, 3-year and 5-year overall survival (OS), respectively, indicating a high prognostic validity (Figure 8A). To further validate that RS had the best prognostic predictive power among clinical variables, we designated these variables as candidate factors and included them in the AUC analysis. The results confirmed our conjecture, with the AUC analysis RS achieving maximum values at 1, 3, and 5 years of OS (Figures 8B–D), which further affirmed the clinical predictive power of the risk signature. Subsequently, combining these clinical variables with RS, a prognostic nomogram was developed for quantitatively predicting the probability of survival at a specific time in HCC patients (Figure 8E). The calibration curve indicated that the prognostic nomogram we developed had reliable predictive performance (Figure 8F).

### 3.8 Association of risk signatures with tumor mutational burden

**FIGURE 9:**
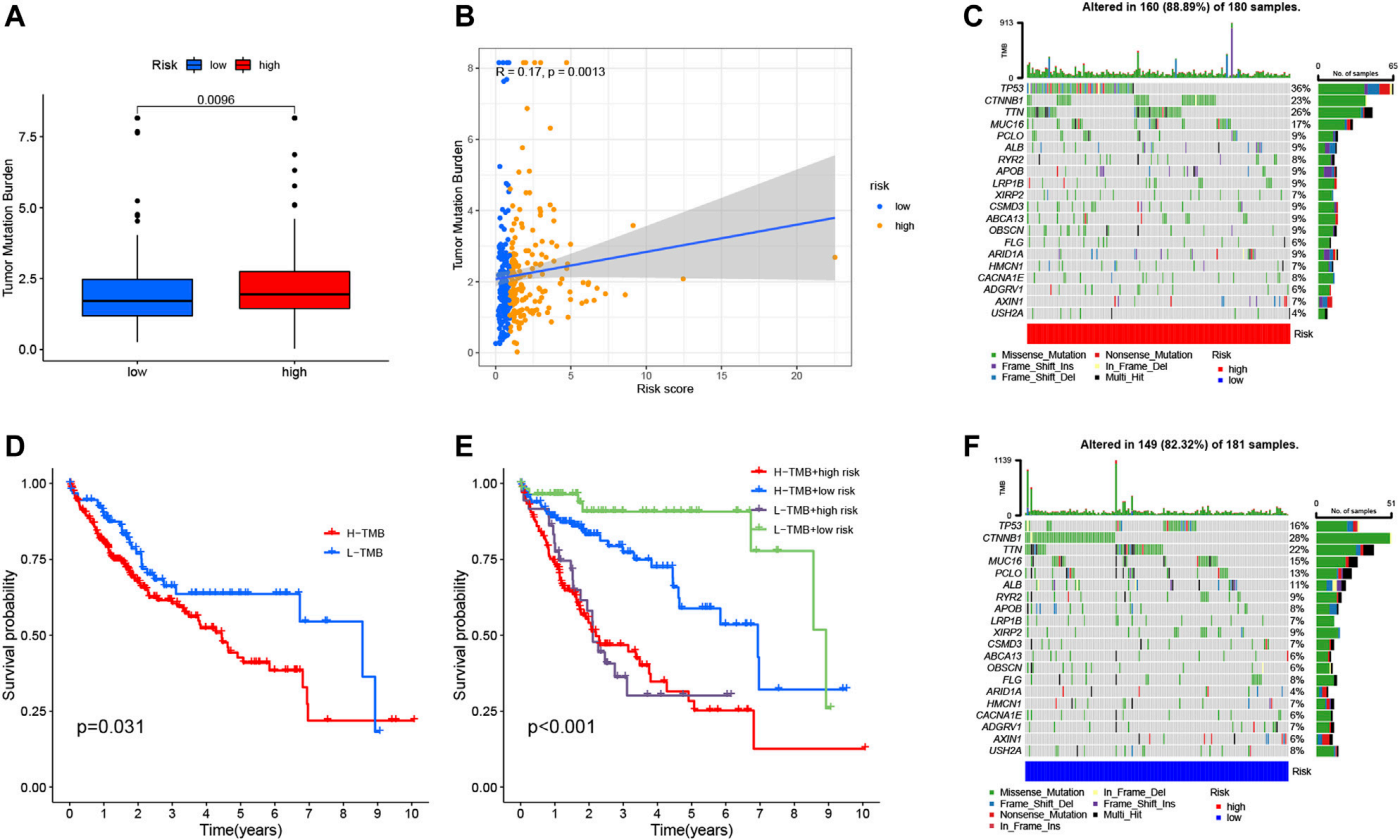
Correlation between risk score and TMB. **(A)** Differences in TMB between HRG and LRG. **(B)** Scatterplots depicting the positive correlation between risk scores and TMB. **(D)** Kaplan-Meier curves of high TMB and low TMB groups. **(E)** Kaplan-Meier curve stratification of patients according to TMB and risk signature. The oncoPrint was constructed using high-risk score **(C)** and low-risk score **(F)**.

Studies have shown that TMB is related to the anti-tumor immune response of immune cells (McGranahan et al., 2016). We speculate that TMB may be an important factor affecting the efficacy of anti-tumor immunotherapy. To this end, we analyzed the differences in TMB in different RS groupings (Figure 9A). Subsequently, we present the distribution of RS and TMB for 370 HCC samples in the form of scatter plots (Figure 9B). It was found that RS and TMB were significantly correlated (R = 0.17, *p* = 0.0013). According to the median of TMB, HCC samples were divided into high- and low-mutation groups, and then KM survival curves were drawn. The results showed that the low-mutation group had a better prognosis compared with the high-mutation group (Figure 9D). The survival curve was drawn according to TMB and RS, and the results showed that the samples with low TMB and low risk had the best survival status (Figure 9E). This also shows that RS and TMB have a certain synergy in predicting HCC survival.

In addition, in order to more intuitively display the somatic mutation situation of the high-risk group and the low-risk group, we drew a comprehensive landscape map of somatic mutations in the high-risk group (Figure 9C) and the low-risk group (Figure 9F). The results showed that genes such as *TP53* (36% vs. 16%), *TTN* (26% vs. 22%), and *MUC16* (17% vs. 15%) had higher mutation rates in the high-risk group, while *CTNNB1* (28%) vs. 23%), *ALB* (11% vs. 9%) and other genes had higher mutation rates in the low-risk group.

### 3.9 Risk signature in tumor immune microenvironment context of hepatocellular carcinoma

**FIGURE 10:**
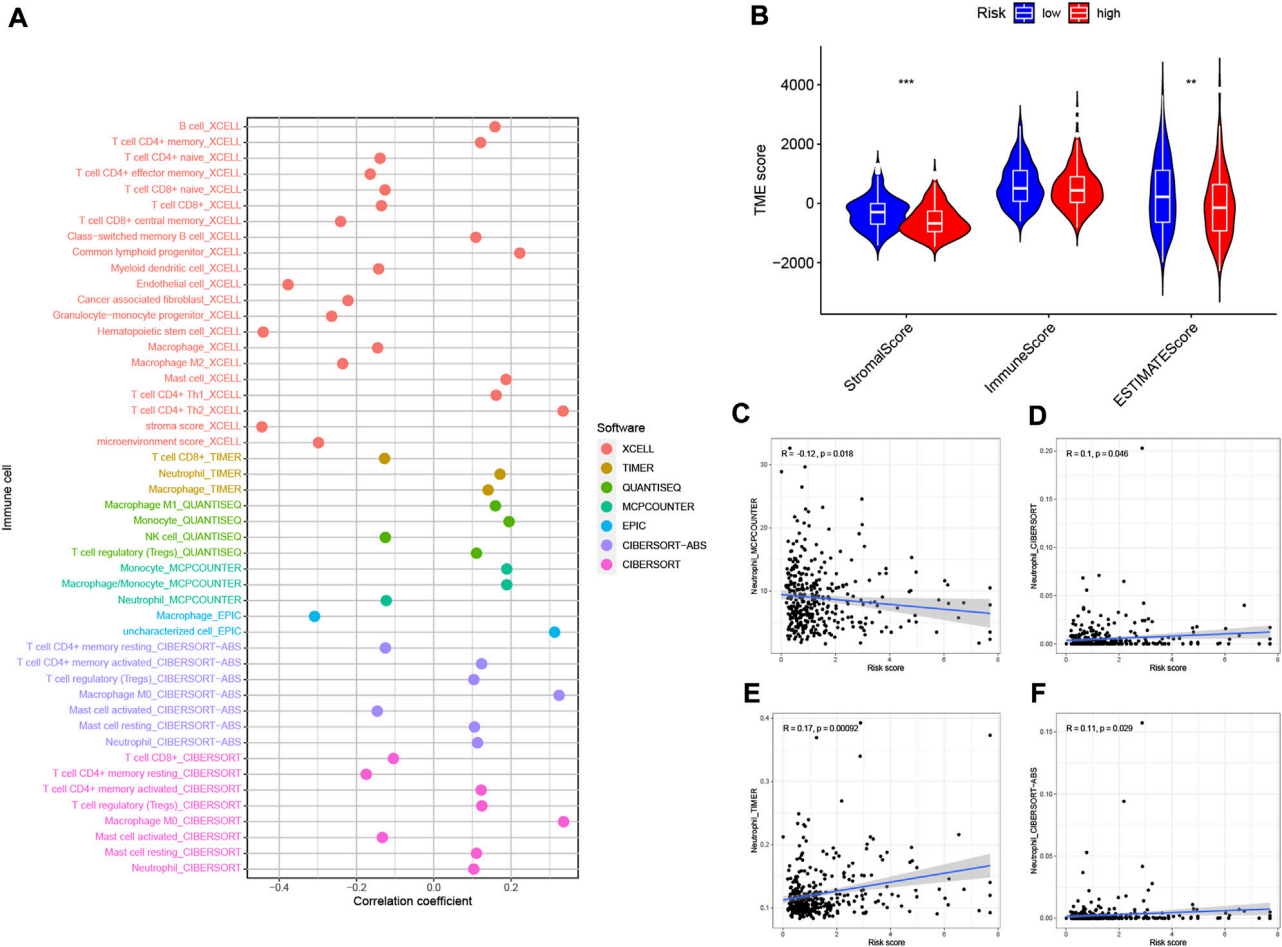
Estimated abundance of tumor-infiltrating cells. Patients in the **(A)** high-risk group had a stronger correlation with tumor-infiltrating immune cells, as shown by the Spearman correlation analysis. **(B)** Association between prognostic risk signatures and central immune checkpoint genes. The correlations predicted by the four methods MCPCOUNTER **(C)**, CIBERSORT **(D)**, TIMER **(E)**, and CIBERSORT−ABS **(F)** were validated.

Based on the intrinsic link between RS and TIME of neutrophil-related genes, we further investigated the contribution of RS to the complexity and diversity of TIME. Using Spearman correlation analysis, the results are shown in Figure 10A (Supplementary Table S5). By ESTIMATE analysis, it was found that the stromalscore and ESTIMATE score showed a significant downward trend in the high-risk group (*p* < 0.01, Figure 10B). Validation of the correlations predicted by the four methods MCPCOUNTER (Figure 10C), CIBERSORT (Figure 10D), TIMER (Figure 10E) and CIBERSORT−ABS (Figure 10F) indicated that our analysis was accurate.

### 3.10 Enriching signaling pathways in low/high risk populations

**FIGURE 11:**
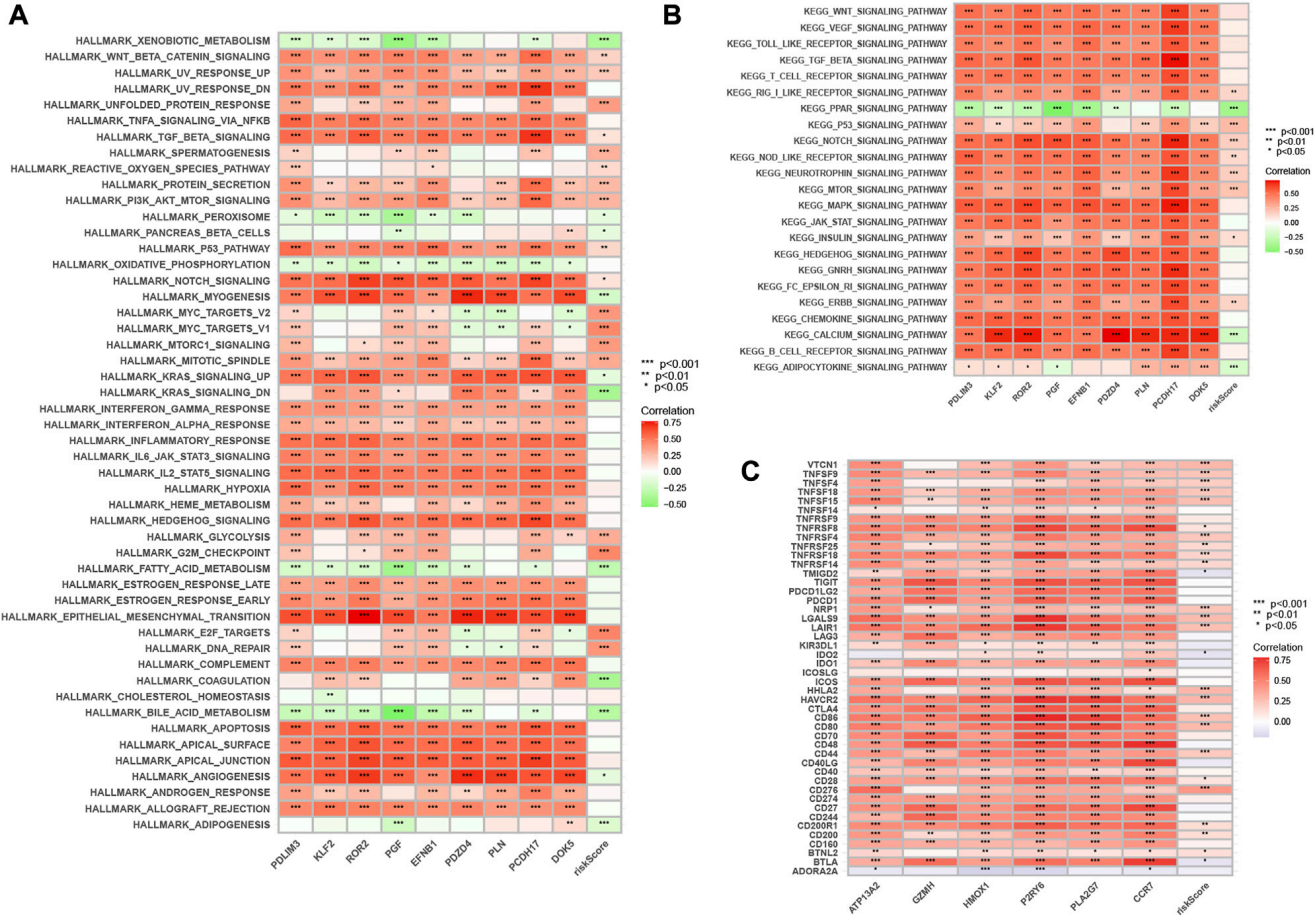
Enrichment pathways of GSVA. **(A)** Heatmap showing the correlation of representative pathway terms of Hallmark with risk score. **(B)** Heatmap showing the correlation of representative pathway terms of KEGG with risk score. Prediction of Immunotherapeutic Response. **(C)** Correlation of expression level of immune checkpoint blockade genes with risk score.

By GSVA analysis (Figures 11A,B), we found that neutrophil-related genes were negatively correlated with KEGG/PPAR signaling pathway and positively correlated with most other signaling pathways. RS is negatively correlated with adipocytokine signaling pathway, and positively correlated with the p53 signaling pathway.

### 3.11 Immunotherapy prediction

**FIGURE 12:**
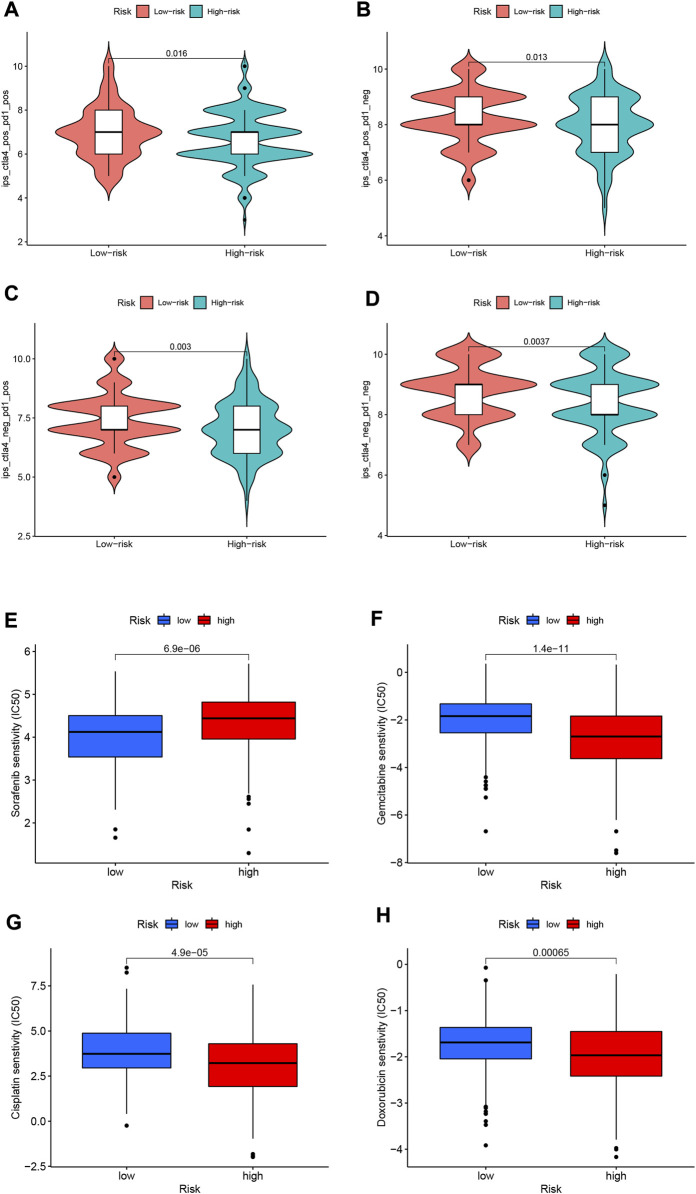
**(A–D)** IPS score distribution map. Estimates of chemotherapy effect risk scores. **(E)** Sensitivity analysis of sorafenib in patients with high and low risk scores. **(F)** Sensitivity analysis of gemcitabine in patients with high and low risk scores. **(G)** Sensitivity analysis of cisplatin in patients with high and low risk scores. **(H)** Sensitivity analysis of doxorubicin in patients with high and low risk scores.

Since there is no information on immunotherapy in the TCGA-LIHC dataset, we used an indirect approach to analyze immunotherapy. The relationship between immune checkpoint blockade-related gene expression and RS was analyzed. The results showed that most of the immune checkpoint blockade-related genes (*VTCN1*, *TNFSF9*, *TNFSF18*, *TNFSF15*, *CD80*, etc.) were positively correlated with the risk score, and a few immune checkpoint blockade-related genes (such as *TMIGD2* and *BTLA*) were associated with RS were negatively correlated (Figure 11C). The IPS scores of different RS groupings are shown in Figures 12A–D. HRG IPS scores were lower when PD1-positive and CTLA4-positive, suggesting that high-risk patients are more suitable for novel immune checkpoint inhibitors (ICIs) immunotherapy.

### 3.12 Predicting response to chemotherapy

Through pRRophetic algorithm analysis, we found that the IC50 of chemotherapeutic drugs (sorafenib, gemcitabine, cisplatin, and doxorubicin) were different in HRG/LRG. We found that sorafenib has a lower IC50 in LRG (*p* < 0.05; Figure 12E), suggesting that sorafenib has a higher drug sensitivity in LRG. In contrast, gemcitabine, cisplatin and doxorubicin had lower IC50s in HRG (*p* < 0.05; Figures 12F–H), suggesting that tumor cells in HRG are more sensitive to these drugs.

## 4 Discussion

The high degree of malignancy of HCC, combined with the generally late diagnosis and inadequate treatment methods, makes it a major threat to human health worldwide (Vanderborght et al., 2020). Treatment is complicated by underlying liver disease in up to 80 percent of all HCC cases (Kirstein and Wirth, 2020). HCC is an inflammation-related malignancy in which TIME can induce immune tolerance and escape through various mechanisms (Fu et al., 2019). As the first barrier against pathogen invasion, neutrophils not only have anti-inflammatory and anti-infection effects, but also play a pivotal role in anti-tumor immunity.

In our study, two datasets, TCGA-LIHC and GSE76427, were used, the former for developing neutrophil-related risk signatures and the latter for external validation. The 10530 genes were divided into 6 gene modules according to their functional similarity using WGCNA and the “dynamic tree cutting” algorithm. The correlation of these 6 gene modules with immune cells in HCC tumor tissues was analyzed by Pearson correlation. The “MEbrown” significantly associated with neutrophils was selected and 590 genes were extracted from this gene module. Through a series of screening, 9 neutrophil-related genes (*PDLIM3*, *KLF2*, *ROR2*, *PGF*, *EFNB1*, *PDZD4*, *PLN*, *PCDH17*, *DOK5*) with good prognostic value for HCC were finally obtained. RS was calculated from the coefficient of each gene and the expression level in the sample. With the median RS as the boundary, all samples were classified as HRG and LRG. We analyzed the survival of HRG/LRG samples in the TCGA-LIHC and GSE76427 datasets, and plotted K-M survival curves. The K-M survival curve plotted using the TCGA-LIHC dataset (Figure 3D) indicated that the LRG sample had a better prognosis (*p* < 0.001). Interestingly, when HRG/LRG sample survival was analyzed using the GSE76427 dataset (Supplementary Figure S1), we found that HRG samples had better prognosis (*p* = 0.041). This may be related to the small sample size in the GSE76427 dataset, which requires sufficient samples for validation.

We used RS to represent neutrophil-related genes to further explore its impact on the prognosis of HCC and its relationship with TMB, cell signaling pathways, immunotherapy and chemotherapy. To display the relationship between risk characteristics and HCC prognosis more intuitively and conveniently, a nomogram was constructed by combining RS and other clinical variables. It can directly use the graph to calculate the value of a variable, such as the patient’s index score or survival probability. In the nomogram model we constructed, if the RS and other clinical variables are known, and the scores obtained by each independent variable are added together, it is possible to predict the 1-year, 3-year year and 5-year survival probability.

We explored the association of risk signature and TMB in this study and found that risk signature and TMB had some synergistic effects in predicting patient survival. Previous studies have found that TMB can be used to predict the efficacy of immune checkpoint inhibitor therapy (Snyder et al., 2014; Hugo et al., 2016; Carbone et al., 2017; Liang et al., 2021; Xu et al., 2022a; Xu et al., 2022b). It has also been found that TMB is associated with the prognosis of diffuse glioma (Wang et al., 2020). Studies have shown that HCC generally has a lower TMB (Ang et al., 2019; Mauriello et al., 2019; Wang and Li, 2019; Li et al., 2020a). In contrast, Ritu and his team found that HCC patients with higher TMB had poorer prognosis (Shrestha et al., 2018). Cai and colleagues found that high TMB was associated with poor prognosis in HCC patients after radical hepatectomy (Cai et al., 2020). These findings further confirm our conclusions.

Among the many treatments for liver cancer, immunotherapy has great advantages. Precise and effective HCC immunotherapy brings a new dawn to patients. In recent years, the clinical application of ICIs has increased the enthusiasm for HCC immunotherapy research. Studies showing that the combination of atezolizumab (anti-PD-L1) and bevacizumab (anti-VEGF) can significantly improve overall survival has made them first-line therapy for patients with advanced HCC (Donisi et al., 2020; Donne and Lujambio, 2022; Sperandio et al., 2022). Besides ICI, there are other immunotherapy strategies under investigation, such as oncolytic virus immunotherapy and adoptive T cell transfer (Foerster et al., 2022). Despite great progress in HCC immunotherapy, current immunotherapies are only able to induce durable responses in a subset of HCC patients (Ruf et al., 2021). There is ample evidence that the effect of HCC tumor immunotherapy is significantly associated with TIME (Riaz et al., 2017; Cheng et al., 2020; Ruf et al., 2021). Immunotherapy of HCC is promising but challenging.

We developed a potential association between neutrophil-related gene-based risk signatures and TIME. It was found that stromalscore and ESTIMATE score had an increasing trend in LRG. This indicates that the tumor purity in the LRG samples is relatively low. Some researchers suggest that TIME can be used as an independent factor affecting the prognosis of HCC patients and provide help for precision medicine (Taube et al., 2018; Xu et al., 2019).

Among the 9 neutrophil-related genes we screened, several genes have been confirmed to play important roles in the occurrence, development and prognosis of HCC tumors. Studies have found that *KLF2* gene plays a tumor suppressor function by inhibiting TGF-β/Smad signaling in HCC cells (Li et al., 2020b). Geng and colleagues found in a study of 85 samples that Ror2 protein deletion was associated with poor prognosis in HCC (Geng et al., 2012). Liu and his team found that PCDH17 is regulated by DNMT3B methylation and inhibits cell proliferation, invasion and migration in HCC *via* EMT (Liu et al., 2022). These existing research results confirm the scientific reliability of our construction of risk characteristics. Although we screened out the 9 genes most related to HCC from many neutrophil-related genes and constructed a prognostic risk model, the underlying mechanisms of these genes’ functions still need to be explored and discovered by a large number of researchers. Of course, our study also has some limitations and needs to be further improved. Therefore, it is necessary to collect tissue samples and validate our results at the cellular, animal and tissue levels separately in the studies noted to make the results more credible.

## 5 Conclusion

In conclusion, bioinformatics-based deciphering of the TIME landscape constructs a prognostic signature dominated by neutrophil-related genes. This prognostic feature has a certain good value in predicting the clinical prognosis of HCC, analyzing gene mutation, TIME heterogeneity and treatment response. Nonetheless, future prospective studies are needed to further examine this feature.

## Data Availability

The original contributions presented in the study are included in the article/Supplementary Material, further inquiries can be directed to the corresponding author.

## Supplementary material

Supplementary Figure S1 K-M survival curves plotted using the GSE76427 dataset.

Supplementary Table S1 The Results of CIBERSORT algorithm.

Supplementary Table S2 The genes and corresponding modules after WGCNA.

Supplementary Table S3 The results of univariate regression analysis.

Supplementary Table S4 The results of multivariate regression analysis.

Supplementary Table S5 The results of correlation of risk score with immune infiltrating cells.

## Abbreviations

HCC, hepatocellular carcinoma; WGCNA, weighted gene co-expression network analysis; ROC, receiver operating characteristic; GSEA, Gene Set Enrichment Analysis; RS, Risk scores; GSVA, Gene set variation analysis; IPS, Immunophenoscore; TIME, tumor immune microenvironment; GEO, Gene Expression Omnibus; TIC, tumor-infiltrating immune cell; HRG, high-risk group; LRG, low-risk group; KM, Kaplan-Meier; TMB, tumor mutational burden; CNV, copy number variation; TCGA-LIHC, The Cancer Genome Atlas-Liver hepatocellular carcinoma; IC50, half-maximal inhibitory concentrations; HR, hazard ratios; AUC, The area under the curve; OS, overall survival; ICIs, immune checkpoint inhibitors.
