# Supplementary figures and images for: Clinical neutrophil-associated genes as reliable predictors of hepatocellular carcinoma
Source: Front Genet. 2022 Oct 6;13:989779. doi: 10.3389/fgene.2022.989779 (PMC9582652; doi:10.3389/fgene.2022.989779)

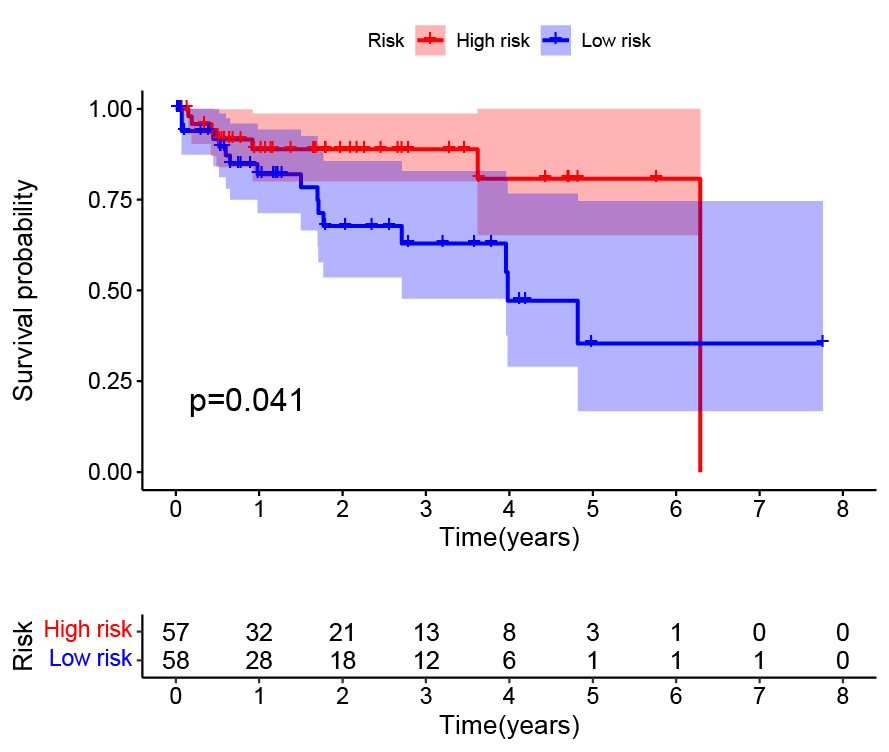

Supplement: Supplementary file 3 [file Image1.TIF]
